# Partial Replacement of Synthetic Vitamin E by Polyphenols in Post-Weaning Piglets

**DOI:** 10.3390/antiox12091752

**Published:** 2023-09-12

**Authors:** Grazia Pastorelli, Rachida Benamri, Massimo Faustini, Roberta De Bellis, Valentina Serra, Lauretta Turin, Marc Haumont, Philippe Durand, Laura Bianchessi, Emmanuelle Prost-Camus, Thomas Pecqueur, Michel Prost

**Affiliations:** 1 Department of Veterinary Medicine and Animal Sciences, University of Milano, Via dell’Università 6, 26900 Lodi, Italy; massimo.faustini@unimi.it (M.F.); valentina.serra@unimi.it (V.S.); laura.bianchessi@unimi.it (L.B.); 2 Cargill Animal Nutrition, Cargill Incorporated, Wayzata, MN 55391, USA; rachida_benamri@cargill.com (R.B.); thomas_pecqueur@cargill.com (T.P.); 3 Department of Biomolecular Sciences, University of Urbino “Carlo Bo”, 61029 Urbino, Italy; roberta.debellis@uniurb.it; 4 Laboratoire Lara-Spiral, 3 rue des Mardors, 21560 Couternon, France; laraspiral@laraspiral.com (M.H.); p.durand@laraspiral.com (P.D.); michelprost.spiral@wanadoo.fr (M.P.); 5 Centre Europeen de Recherche et Analyses, 3 rue des Mardors, 21560 Couternon, France; centreeuropeen@orange.fr

**Keywords:** alpha-tocopherol, weaned piglets, dietary polyphenols, antioxidant status, immunity, cytokines

## Abstract

Vitamin E is an essential nutrient usually recommended in post-weaning piglets, when a decline in the serum vitamin E concentration is observed. Selected polyphenols have the potential to partially replace vitamin E in animal feed. The aim of this study was to investigate the effect of the dietary inclusion of some commercial polyphenol products (PPs) on the growth performance, antioxidant status and immunity of post-weaning piglets. A total of 300 piglets (BW 7.18 kg ± 1.18) were randomly assigned to six dietary groups: CON^−^ (40 mg/kg vitamin E); CON^+^(175.8 mg/kg vitamin E); and PP1, PP2, PP3 and PP4, in which 50% vitamin E of CON^+^ was replaced with PP with equivalent vitamin E activity. The PP1 group exhibited lower performance (*p* < 0.05) than the other dietary groups, but a similar performance to that commonly registered in pig farms. Dietary polyphenols did not influence the IgG concentration or the IL-6, IL-10, IFN-γ and TNF-α cytokine concentrations. A lower IL-8 level was found in the PP4 group than in the other groups. The diets that affected the vitamin A content showed the highest value (*p* < 0.05) in the PP1 group, and a trend was noted for vitamin E with a higher content in PP4 and CON^+^. The polyphenols-enriched diets, especially the PP3 diet, maintained an antioxidant capacity (whole blood KRL) similar to the CON^+^ diet. In conclusion, the replacement of vitamin E with all PPs enables partial vitamin E substitution in post-weaning piglets.

## 1. Introduction

Vitamin E (tocopherol) is an essential lipid-soluble micronutrient known for its antioxidant, anti-inflammatory and immunomodulatory properties [1,2]. In feeding experiments with weaned pigs, several authors have observed a drop in vitamin E concentrations in the plasma in the first weeks after weaning [3,4]. Various factors influence the pig’s vitamin E status both before and after weaning. Neonatal pigs are born with low tissue reserves of α-tocopherol [5]. Although colostrum contributes α-tocopherol to the neonate, its concentration is dependent on the dietary vitamin E level fed to the sow. In neonatal pigs administrated iron (Fe), serum α-tocopherol declines, as if vitamin E was used to curtail the pro-oxidant activity of injected Fe. Moreover, weaning causes a dramatic decrease in the activity of carboxyl ester hydrolase, the enzyme that cleaves the dl-α-tocopheryl acetate form of the vitamin used in standard commercial feeds. Adequate plasma vitamin E levels in weanling pigs may be attained with sufficient dl-α-tocopheryl acetate in the feed [6,7].

Furthermore, in order to prevent this deficiency in nursery pigs, the supplemental vitamin E has frequently been increased to 200 IU/kg, a level that is substantially higher than the 16 IU vitamin E/kg diet suggested by the National Research Council (NRC, ref. [8]).

Actually, the NRC (2012) recommends 16 and 11 UI/kg for piglets in the range of 7–11 and 11–25 kg, respectively. Supplementation with high levels of vitamin E is usually recommended for diets used during the post-weaning period, when piglets show reduced growth rates and are more susceptible to disease [9].

Improvements in the cell-mediated and humoral immune responses have been reported in various animal species by adjusting the dietary levels of vitamin E [2]. In particular, vitamin E supplementation resulted in increased lymphocyte proliferation, immunoglobulin levels, antibody responses, natural killer (NK) cell activity and interleukin (IL)-2 production. [2].

In recent years, there has been a growing level of awareness among food manufacturers regarding the origin of feed additives in livestock feed; consequently, the request for products of natural origin continues to increase. Polyphenols are substances of plant origin that have antioxidant properties [10], and may reduce the negative effects of oxidative stress [11,12]. Their antioxidant potential is comparable with that of the major biological antioxidants: tocopherol and ascorbic acid [13]. Polyphenols also have immunomodulatory, anti-inflammatory and bactericidal properties [14], making their use crucial in the post-weaning period. For example, polyphenolic compounds like curcumin and resveratrol have exhibited many beneficial effects in 21-day-old weaned piglets, by alleviating intestinal inflammation and improving intestinal immune function [15]. The dietary supplementation of grape pomace improved the intestinal microbiota and down-regulated the expression of pro-inflammatory cytokines in post-weaning piglets fed for 4 weeks [16]. Other studies reported beneficial effects of dietary polyphenols on the growth performance of weaned piglets [17,18].

This research postulated that polyphenols can partially replace vitamin E in terms of antioxidant activity based on the assumption of having a 50% equivalency to vitamin E (DL-α-tocopherol acetate).

The aim of the present study was to investigate the effects of partially replacing vitamin E with different commercial polyphenol products on the growth performance, antioxidant defenses and immune response of piglets during a 35-day post-weaning period.

## 2. Materials and Methods

### 2.1. Ethics Statement

Our in vivo trial complied with Italian regulations on animal experimentation and ethics (Legislative Decree 26/2014) [19], in accordance with European regulations (Directive 2010/63) [20], and was approved by the Animal Welfare Body of the University of Milan (number 140/2021). The study was performed between April and May 2022 at the Productive Pig Unit “Cascina Agrieffe” farm located at Gottolengo (BS) Lombardy (Italy).

### 2.2. Animals and Treatments

A total of 300 crossbred piglets (Large White x Landrace), (28 days old ± 2 days), with a mean body weight (BW) of 7.18 kg ± 1.18, were selected from 30 litters of contemporary sows. The piglets were individually ear-tagged and divided into six experimental dietary groups (5 pens per diet, 10 piglets per pen), balanced for sex and body weight. Each pen (2.0 × 1.5 m) was equipped with a self-feeder and nipple drinkers to allow ad libitum access to feed and water during the 35-day experimental period. The rooms had a forced-air ventilation system set at 60% relative humidity, and a temperature of 27 ± 2 °C. Each pen was provided with metal chains, with soft wooden bars hanging from the walls and straw-filled baskets as enrichment material.

The animals were assigned to six dietary treatments: a negative control diet (CON^−^) corresponding to a diet with a low vitamin E content (40 mg/kg of vitamin E); a positive control diet (CON^+^) corresponding to the standard diet used by farmers in which the vitamin E content (175.8 mg/kg) exceeds the nutritional requirements as recommended by NRC (2012 [8]); polyphenol product (PP) diets in which four different commercial polyphenol products (PP1, PP2, PP3, PP4) with equivalent vitamin E activity in terms of antioxidant capacity replaced 50% vitamin E of CON^+^ (87.9 mg/kg vitamin E). The tested commercial polyphenol products contained the following:

PP1: Mix of citrus, grape and chestnut extracts and carrier;
PP2: Dried grape extract and carrier;
PP3: Freeze-dried melon juice and flesh palm oil and microcrystalline cellulose;
PP4: Grape and onion soluble, flavoring compounds and carrier.

The experimental diets were formulated to be isoenergetic and isoproteic on net energy, and were produced with the same batches of feeds by Tracciaverde S.R.L. (Bonemerse, Italy). None of the experimental diets contained any antimicrobial or growth promoter, and all were designed to meet or exceed the nutrient requirements of weaned piglets recommended by the NRC (2012 [8]; Table 1).

### 2.3. Growth Performance

The piglets were individually weighed on day 0 (d0) and on day 35 (d35), and the pen feed consumption (experimental unit for the feed intake evaluation) was recorded. The feed conversion ratio (FCR) was calculated by dividing the amount of feed consumed during the experimental period by the growth of the animals during that same time. The average daily gain was calculated from the measurements of weight and the number of experimental days. The feed intake of the pen was calculated by the difference between the offered feed and leftovers. The leftovers, if any, were weighed daily and considered for the final calculation of the feed consumed. The mortality was recorded daily throughout the trial.

### 2.4. Collection of Blood Samples

At the beginning (d0) and at the end of the dietary trial (d35), blood samples were collected from 2 randomly selected male piglets per pen via jugular vein puncture before the morning feeding (total number of specimens = 50).

Vacuum tubes (9 mL) containing K3EDTA (Vacuette^®^ Tube, Cat. no. 455036; Greiner Bio-One, Kremsmünster, Austria) for plasma collection, and 9 mL vacuum tubes for serum (Vacuette^®^ Tube, Cat. no. 455092; Greiner Bio-One, Kremsmünster, Austria) were used.

After collection, the blood samples were immediately transported to the laboratory. Plasma and serum were obtained from the blood samples by centrifugation (3500× *g* for 15 min at 4 °C), and were stored at –18 °C until subsequent analyses.

### 2.5. Blood Analyses

#### 2.5.1. Vitamin A and Vitamin E Analysis

The retinol and tocopherol concentrations were measured via chromatography (HPLC) (Shimadzu, Japan) according to the protocol reported in Rettenmaier and Schüep, 1994 [21] and through an iCheck fluorometer–spectrophotometer (iCheck Vitamin E; BioAnalyt GmbH, Teltow, Germany) as described in Simoni et al., 2022 [22], respectively.

#### 2.5.2. IgG and Cytokines Analysis

The serum IgG concentration was determined using a commercial enzyme-linked immunosorbent assay (ELISA; Porcine IgG (Immunoglobulin G) ELISA Kit; FineTest, Wuhan Fine Biotech Co., Ltd., Wuhan, China; cat. no. EP0084) based on sandwich binding. The colorimetric reaction was catalyzed with streptavidin-conjugated horseradish peroxidase (HRP), which produced a yellow product that was proportional to the target amount present in the sample. The serum samples were diluted at 1:200,000 before being analyzed in duplicate. The range of detection for this ELISA assay was 1.563–100 ng/mL.

The serum cytokines (IL-6, IL-8, IL-10, INF-γ) concentrations were determined with Luminex technology (Labospace S.r.l., Milan, Italy); the Luminex test allowed for quick and accurate measurements of the given targets using hundreds of internally colored plastic microbeads with a graduated mixture of red or infrared fluorescent dyes that emitted light at different wavelengths when struck by a laser [23].

The TNF-α levels were measured using a commercial sandwich ELISA assay (Cloud-Clone Corp., Katy, TX, USA; cat. no. SEA133Po) with a detection range of 15.6–1000 pg/mL. The wells of the microplate were pre-coated with antibodies specific to TNF-α and, after the addition of samples, avidin-conjugated to HRP, and the TMB chromogen substrate exhibited a color change that was measured spectrophotometrically at a wavelength of 450 nm.

#### 2.5.3. Antioxidant Defenses

The activity of glutathione peroxidase (GPx) was determined using a commercial assay kit (Cayman Chemical Co., Ann Arbor, MI, USA, cat. no. 703102) according to the instructions provided by the manufacturer. Oxidized glutathione (GSSG), produced during the reduction of hydroperoxide by GPx, is recycled to its reduced state (GSH) by glutathione reductase (GR) and NADPH. The oxidation of NADPH to NADP+ is accompanied by a decrease in absorbance at 340 nm. Under conditions where GPx activity is limiting, the rate of decrease in absorbance is directly proportional to the GPx activity in the sample. One International Unit (U) is defined as the amount of GPx that will cause the oxidation of 1.0 nmol of NADPH to NADP+ per min at 25 °C.

Superoxide dismutases (SODs) catalyze the dismutation of the superoxide anion into hydrogen peroxide and molecular oxygen. There are three forms of human SOD: cytosolic Cu/Zn-SOD, mitochondrial Mn-SOD and extracellular SOD. The latter is present in plasma. The SOD activity was determined using a commercial assay kit (Cayman Chemical Co., cat. no. 706002) following the manufacturer’s instructions. A water-soluble tetrazolium salt is used to detect the superoxide radical generated by the oxidation of xanthine into uric acid by xanthine oxidase. Superoxide ions that have not been removed by the superoxide dismutase present in the sample react with the tetrazolium salt to form a formazan dye. One International Unit (U) is defined as the amount of SOD needed to exhibit 50% dismutation of the superoxide radical. The 50% inhibitory activity by SOD can be determined using a colorimetric method, measuring the absorbance at 450 nm.

The overall antioxidant defense potential was determined with the biological KRL™ Test (M. Prost Patent) [24,25]. Whole blood and erythrocytes are submitted to oxidant stress, and the free radical-induced hemolysis is recorded via optical density decay with the KRL microplate reader. Inside the body, both the extracellular and intracellular antioxidant defense contribute to maintaining cellular integrity until hemolysis. The resistance of whole blood (KRLWB) and red blood cells (KRLRBC) to free radical attack is expressed as the time that is required to reach 50% of maximal hemolysis (half-hemolysis time, T1/2 in minutes).

### 2.6. Antioxidant Activity of Diets

#### 2.6.1. Sample Treatment

Finely ground samples of all diets were extracted by adding 2 g of each sample to 10 mL of 70% (*v/v*) ethanol/double-distilled water. All samples were shaken in the dark for 1 h and then centrifuged at 13,000 rpm for 15 min at 4 °C. The recovered supernatants were stored at −20 °C until the analyses were carried out.

#### 2.6.2. Antioxidant Activity (ORAC Assay)

The antioxidant capacity of the experimental diets was measured in terms of the Trolox equivalent antioxidant capacity, using the oxygen radical absorbance capacity (ORAC) assay. The assay was performed as in De Bellis et al., 2019 [26], detecting the fluorescence until total extinction (485 nm ex. and 520 nm em.) on a Fluostar Optima plate reader (BMG Labtech, Offenburg, Germany).

The results were compared to the Trolox antioxidant capacity, and, for this reason, the data are reported as the micromoles of Trolox equivalents (µmol TE/mg) of the samples. All reaction mixtures were prepared in duplicate, and at least three independent assays were performed for each sample.

### 2.7. Statistical Analysis

Statistical analyses were performed using SPSS 26.0 (SPSS Inc., Chicago, IL, USA). Before hypothesis testing, all data were examined for normality and transformed where appropriate. The growth performance data were analyzed using the ANOVA procedure, with treatment and sex as the main effects, and the pen was considered as the experimental unit. Parameters for the blood samples (where multiple data were available for each animal) were subjected to repeated analyses. Vitamin E and TNF-α were natural-log-transformed to meet the normality assumption, and therefore subjected to analysis. The treatment effects were deemed significant at *p* < 0.05, and a trend was noted when the *p*-values were between 0.05 and 0.1.

## 3. Results

### 3.1. Antioxidant Activity of Diets and Growth Performance

**Figure 1:**
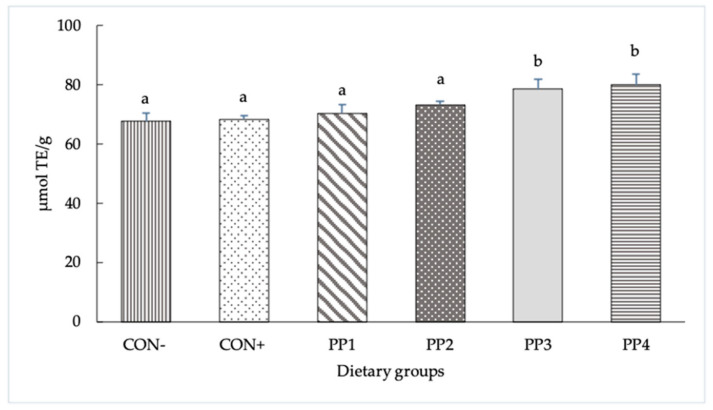
Antioxidant activity levels of experimental diets measured with ORAC assay. The data are reported as means ± SD. ^a,b^ Bars with different letters are significantly different (*p* < 0.05).

The antioxidant activity levels of the six experimental diets measured using the ORAC assay are reported in Figure 1. The ORAC values of the experimental diets showed a significant difference between groups (*p* < 0.001), with concentrations equal to 67.8, 68.4, 70.5, 73.3, 78.6 and 80.0 µmol TE/mg in the CON^−^, CON^+^, PP1, PP2, PP3 and PP4 groups, respectively, with PP3 and PP4 showing the highest values.

Concerning the growth performance, the average BW of the piglets at the beginning of the study was 7.18 kg, without differences due to dietary treatments and sex (7.12 kg female vs. 7.25 kg males) in accordance with the experimental design (*p* > 0.05) (Table 2).

However, at the end of the trial, after 35 days of differentiated feeding, the PP1 group exhibited a lower (*p* < 0.05) final body weight with a lower (*p* < 0.05) average daily gain (ADG) and worse feed conversion ratio (FCR) than the other six dietary treatments. No differences among the CON^+^, CON^−^, PP2, PP3 and PP4 groups for final body weight, ADG and FCR were found. At the end of the trials, there was no difference found between males and females (18.97 vs. 18.47). Moreover, all these results were not affected by health problems. In this regard, no mortality was registered throughout the entire period.

### 3.2. Blood Parameters

Table 3 reports the effects of dietary treatment at two different sampling times on the blood parameters of the piglets throughout the experiment.

At d0, just before the pigs were assigned to receive experimental diets, there was no difference among the treatments for any studied variable (*p* > 0.10). At the end of the trial (d35), the plasma concentrations of vitamin A were greater in the PP1 and CON^+^ groups compared to others (*p* < 0.05), with the highest positive variations observed in the PP1 group. Concerning the diet effect, the serum parameters evaluated were not significantly different among the treatments (*p* > 0.05), except for vitamin A and IL-8 (*p* < 0.001). For the vitamin E values, it was observed that piglets fed a CON^+^ diet reached, on average (average of value at d0 and d35), higher values in comparison with other dietary treatments (1.45 vs. 0.92, 0.97, 0.97, 1.17 and 1.09 for CON^−^, PP1, PP2, PP3 and PP4, respectively). The sampling time significantly affected the vitamin E content, with the lowest levels at day 0 for all groups. The vitamin E variation (%) result was significant (*p* < 0.05), with the highest positive value in the PP4 group vs. other the dietary treatments. The IL-8 results showed a time effect (*p* < 0.001) and a treatment effect (*p* = 0.022) with increased IL-8 at day 35. The comparison post hoc tests showed the following significances: CON^−^ vs. PP2, CON^−^ vs. PP4, CON^+^ vs. PP2 and CON^+^ vs. PP4.

### 3.3. Antioxidant Defenses

Table 4 shows the effects of the dietary treatment and sampling time on the antioxidant defenses of the piglets throughout the experiment.

Concerning plasma antioxidant enzyme activities, the dietary sources significantly affected the plasma GPx activity, which was higher after 35 days (*p* < 0.001). A Tukey post hoc test revealed significant pairwise differences between group PP1 and other groups for GPx (*p* < 0.043). We found that the GPx activity was especially increased in this diet group, rising from 0.96 ± 0.20 to 2.33 ± 0.39 U/mL between d0 and d35.

The overall plasma SOD activity decreased significantly between d0 and d35. Although the lower initial SOD values in groups PP2 and PP4 make results interpretation difficult, we found that the SOD activity after 35 days was significantly higher with diets supplemented with polyphenols PP2, PP3 and PP4 than in the CON^−^ and CON^+^ diets (Mann–Whitney *p* < 0.05).

Our results also show that the dietary source and time significantly affected the antioxidant defense potential measured in whole blood (*p* < 0.05). They show that whole blood KRL decreased significantly between d0 and d35 in the CON^-^ and PP1 groups, but was more stable (paired *t* test *p* > 0.05) in the other groups. At d35, whole blood KRL was higher in the CON^+^ and PP3 diets than in the CON^−^ diet (Mann–Whitney *p* < 0.05). Moreover, a positive correlation was observed between the whole blood antioxidant defense potential and plasma SOD activity in piglets after 35 days (Pearson correlation, r = 0.3248 *p* = 0.011). No significant variations were found in the potential of antioxidant defenses measured at the level of red blood cells.

## 4. Discussion

Post-weaning is a critical stage for the health of piglets, as it affects the feed intake, immunity and redox status of the animals. The different diets tested were formulated to verify the effect of partially substituting the vitamin E content with different polyphenol products on the growth performance and some blood indicators mainly related to immunity and antioxidant status.

In the current study, the feed antioxidant capacity was measured using the ORAC assay, accepted as the current food industry standard for evaluating the antioxidant capacity of food additives, whole foods, juices and raw vitamins [27]. The higher ORAC values found both in the diets supplemented with PP3 and PP4 indicate a greater antioxidant activity compared to the other PPs, and even more than control groups; this is attributable to the presence of polyphenols such as flavanones (grapes), flavonols (onions), phenolic acids and flavonoids, which were mainly concentrated in grape seeds and skins [28] in the second group mentioned (PP4).

The zootechnical performance of piglets can be considered an indirect indicator of animal health. The mechanism of action of polyphenols as growth promoters lies in the fact that they can increase the secretion of endogenous enzymes, bile, mucus and salivary glands, retard the growth of pathogenic microorganisms in the gastrointestinal tract, and modulate gut morphology and architecture through their immunostimulatory, anti-inflammatory and antioxidant functions [29,30]. Furthermore, studies have shown that the utilization of polyphenolic compounds from aromatic plants in animals improved feed intake and growth performance, due to their enhancing effect on the flavor and palatability of feeds [31].

In our study, no differences were observed among the treatments (CON^−^, CON^+^, PP2, PP3 and PP4) regarding feed intake, ADG and FCR. The growth performance results of the PP1 group are probably attributable to the composition of the supplement that likely yielded unpleasant tastes and odors such as to modify the intake, presumably due to the presence of chestnuts that contain tannins, and citrus fruits. In fact, the bitter taste typical of citrus fruits seems to reduce palatability [32]; another study [33] also confirmed that bitterness is the main reason for the rejection of various food products.

Zhang et al. 2014 [34] reported no effect of diets in piglets fed for 21 days with a mixture of standardized plant extracts containing apple (16.5%), grape seed (27.5%), green tea (30%) and olive leaves (2.5%).

Furthermore, Gessner et al. [35] did not show significant differences in growth performance in a study on post-weaning piglets supplemented with 1% polyphenols derived from grape seeds and pomace meal [35]. Conversely, Rajković et al. [36], showed a final live weight (25.4 kg) that was higher (*p* < 0.08) than the control group (23.8 kg) in newly weaned piglets (6.9 kg) fed for 56 days with a diet based on corn supplemented with grape extracts (150 g/t) [36].

In showing similar results, the three vitamin E replacement strategies represented by PP2, PP3 and PP4 suggest that polyphenols can likely replace vitamin E; surprisingly, the CON^−^ group reached a similar growth performance.

In general, the growth performance of piglets in our study reported values that are commonly registered in commercial pig farms, confirming good management, especially considering that the feed was not medicated. No mortality underlined good health conditions of the piglets; therefore, no metabolic shifts were needed to redistribute nutrients away from the growth processes toward immune system function, with a subsequent decrease in feed efficiency for growth.

Vitamins E and A are both antioxidant molecules; vitamin E is mainly related to hydroperoxyl radical scavenging [37], while vitamin A acts by donating hydrogen atoms [38]. The greater vitamin A concentration in pigs fed PP1 during post-weaning may be indicative of a lower inflammatory status, and in this study, it seems to be associated with greater GPX activity. The addition of PP1 to diets led to an increase (by 30% on average, *p* = 0.022) in serum retinol levels in comparison with the remaining groups.

The highest content of vitamin A in blood from piglets in the PP1 group at d35 could be related to the accumulation action from carotene content and antioxidant compounds from the dietary supplement; the citrus part contains polyphenols, primarily flavonoids. We speculated that the increased vitamin A level could be attributed to the ability of polyphenols of PP1 to strengthen and save the endogenous antioxidant system, as reported by Corbi et al. in rabbits [39].

Even though vitamin E and polyphenols are both added to swine diets for antioxidant purposes, they have a different mechanism of action. Vitamin E can be absorbed in the intestine and enter the systemic circulation, as literature reported that supplementing vitamin E in pig diets increased serum and tissue (loin muscle, liver, and fat) vitamin E concentrations [40,41,42,43]. On the other hand, there have been few studies conducted in vivo on the digestibility and bioavailability of polyphenols in pigs. Research studies suggest that only low percentages of dietary polyphenols may be absorbed in the small intestine, and have a low bioavailability due to their molecular structures [14,44,45]. The polyphenols are expected to have direct antioxidant effects in vivo in the intestinal lumen because of the higher concentration of polyphenols in the lumen compared to the systemic concentrations [46]. Moreover, the low amounts of absorbed polyphenols are then extensively bio-transformed in the liver and rapidly excreted in the urine and bile [47]. Subsequently, the colon microbiota’s enzymes transform the bile-excreted polyphenol metabolites and the unabsorbed polyphenols into various metabolites [46,48]. The present study was not focused on the microbiota; therefore, we could not evaluate this aspect.

No clinical sign of vitamin E deficiency was noted in any of the piglets. In general, the raw data are in line with the concentrations detected in piglets in the same physiological stage [42], and also agree with the time effect found by Moreira and Mahan [49], who registered an increase from day 21 to day 42. Our trial ended 35 days after weaning. A vitamin E dose of 175.8 mg/kg increased the total tocopherol content in the blood serum, whereas the CON^−^ diet was the lowest on average, as expected.

There is no general agreement in the literature regarding the limits between adequate, marginal and deficient plasma vitamin E levels in pigs [50]. As reported by Sivertsen [50], the National Veterinary Institute has considered plasma levels of vitamin E below 1.0 μg/mL as distinctly deficient, while normal is considered equal to 2.0 mg/L [51]. It should be underlined that the proportion of pigs with a plasma vitamin E level below 1.5 μg vitamin E/mL is very common [52,53].

The IgG results showed a time effect (*p* < 0.05) that is in agreement with the literature. This immunoglobulin isotype, which is the most important and abundant defense molecule against infections, has been reported to decrease in concentration after weaning at the time of depletion of maternal immunity [54,55], and subsequently increases as an effect of the beginning of piglets’ own synthesis of IgG, which starts at day 7, increases up to day 28 [56] and later up to day 45 [57], according to different studies. In the present study, which ended at day 35, an increase in IgG was observed already at such a time point. Dietary polyphenols did not influence the IgG concentration, confirming the data from Pistol et al. [58].

It is well-recognized that multiple factors that occur during weaning can lead to oxidative stress in post-weaned piglets.

Oxidative stress changes the structure of biomolecules, such as proteins, lipids and nucleic acids, leading to cell or organ injury; in addition, it may change gastrointestinal functions and induce cell apoptosis [59]. It may result in growth restriction, disease and even death [60].

Inflammation is the consequence of oxidative stress, and the pathways which produce the mediators of inflammation, such as cytokines, are all induced by oxidative stress [61]. Cytokines, including pro-inflammatory cytokines and anti-inflammatory cytokines, are necessary mediators of inflammatory responses. The balances of cytokines are central for protection against or susceptibility to infections [62]. Early life development is a stressful period for pigs, as the immature intestines of young animals are very vulnerable to invading pathogens, leading to inflammation [63]. Pro-inflammatory cytokines such as TNF-α, IL-6 and IL-8 trigger inflammatory responses and have negative effects on intestinal integrity and epithelial function [64]. In contrast, anti-inflammatory cytokines such as IL-10 hinder the inflammatory response [65]. Different studies found significant reductions in pro-inflammatory cytokines, such as TNF-α, NF-kB, IL-1β, IL-1ra, IL-2, IL-4, IL-6 or IL-8, when vitamin E [66] or polyphenols were fed to the nursery pigs [58,67,68].

In the current study, no statistically significant difference in anti-inflammatory cytokine (IL-10) was found among the dietary PP groups. Although no statistically significant differences were found, with the exception of IL-8 in the PP4 group, some pro-inflammatory cytokines showed a reduction at d35 in pigs fed diets formulated with PP4 (IFN-γ TNF-α), PP1 and PP2 (IL-6). A reduction in pro-inflammatory cytokines levels in healthy pigs indicates an improvement in immune status, which suggests that these pigs may be able to spend less energy on activating the immune defenses, which can potentially lead to improved energy utilization [41] and increased performance. However, in the present study, the growth performance results seem to be independent of the cytokines results.

All cytokine data are in the range found for piglets at the same age [69,70]. A significant time effect was detected for IL-8, TNF-α and IFN-γ (*p* < 0.05), showing higher values at Time 2 in comparison to Time 1, according to a previous study [71]. Conversely, the levels of IL-6 and IL-10 did not show any significant value in considering the time or the treatment effect. The pro-inflammatory cytokine IL-8 was significantly lower in the PP3 and PP4 groups than in the positive and negative controls. This may be explained by a diminished need for such chemoattractant cytokines to be functioning in the recruitment of neutrophils and other inflammatory cells.

Previous studies have shown a close relationship between post-weaning stress syndrome in piglets and oxidative stress that can last for weeks [72]. Whole blood KRL results emphasize that the overall potential of antioxidant defenses decreases after a 35-day post-weaning period, especially in piglets with a low vitamin E diet (CON^−^). Although this is not significant, they also suggest that this decrease would be partly normalized by vitamin E or vitamin E/polyphenol supplementations, as in the CON^+^ and PP3 groups. These restorations of antioxidant defense potential would be associated with an increase in superoxide dismutase activity that was observed in piglets supplemented with the PP2, PP3 and PP4 diets.

SOD and glutathione peroxidase (GPx) represent the main antioxidant enzymes in mammals, and protect the organism against prooxidants by reducing the accumulation of organic hydroperoxides and hydrogen peroxide. The activity of these enzymes is commonly used to monitor the antioxidant capability of the body [73].

Our analyses underline that plasma glutathione peroxidase activity increases in all piglets after 35 days of treatment, especially in animals with a PP1 diet. This increased plasma GPx activity in piglets suggests the induction of antioxidant defense mechanisms; in fact, it has been reported that antioxidant enzymes prevent the hosts from oxidative stress by increasing their activities [74]. This increase in plasma GPx activity is associated with a rapid increase in antioxidant demand, and has been described in other studies. [75].

Concerning the economic impact of this experimental trial, it should be underlined that apart from PP3, all tested commercial products have an inclusion price that is lower compared to synthetic vitamin E; therefore, the cost of the feed including the PP1, PP2 and PP4 products is lower than that of the CON^+^ treatment. On the other hand, as the PP1 treatment decreased performance of piglets compared to the CON^+^ treatment, the economic impact of this product is of negligible importance. Based on the obtained results, as PP4 treatment is less expensive than vitamin E and the results are positive, we can assume that the chances of obtaining a positive economic impact with this commercial product are very likely to occur.

## 5. Conclusions

The present study on the partial replacement of vitamin E in the diet of weaned piglets with different polyphenol products provides interesting results, since they come from a period characterized by high vitamin E requirements.

All experimental diets had no negative effects on the growth performance of piglets in the post-weaning period.

Replacing 50% of the vitamin E of the positive control diet with PP1 improved the vitamin A concentration and the antioxidant activity measured as glutathione peroxidase levels.

PP4 seems to be the most promising integration for the plasmatic increase in alpha tocopherol due to the spare action exerted by polyphenols, and contributed to a lower pro-inflammatory activity of IL-8.

Hence, under farming conditions among the polyphenol products, the PP4 treatment may be the most advisable integration as a result of its better growth performance observed compared to PP1.

The results of this study will have to be confirmed by further research, which should be extended to the subsequent growth phase for the evaluation of long-term effects.

## Figures and Tables

**Table 1:** Ingredients (%) and composition of basal diets (as-fed basis).

Ingredients as % of Feed Basis	CON^−^	CON^+^	PP
Barley meal	25.90	25.87	25.56
Wheat meal	22.45	22.45	22.45
Soy protein concentrate fermented	7.0	7.0	7.0
Flaked corn	7.0	7.0	7.0
Corn meal	5.0	5.0	5.0
Flaked wheat	8.89	8.89	8.89
Whey powder	4.0	4.0	4.0
Dextrose monohydrate	3.0	3.0	3.0
Hulled flaked barley	3.0	3.0	3.0
Soy protein concentrate	5.0	5.0	5.0
Soybean oil	1.5	1.5	1.5
Dried sugar beet pulp	1.5	1.5	1.5
Acidity regulators	1.0	1.0	1.0
Coconut oil	1.0	1.0	1.0
Plasma	1.0	1.0	1.0
L-Lysine	0.6	0.6	0.6
Fish meal	0.5	0.5	0.5
PP1, PP2, PP3, PP4	-	-	0.330
DL-methionine	0.23	0.23	0.23
Dicalcium phosphate	0.20	0.20	0.20
Calcium carbonate	0.2	0.2	0.2
L-treonine	0.2	0.2	0.2
Vitamin mineral premix ^1^	0.20	0.20	0.20
Salt	0.15	0.15	0.15
L-valine	0.11	0.11	0.11
L-tryptophane	0.04	0.04	0.04
Aromas	0.08	0.08	0.08
Vit E supplement ^2^	0.00	0.028	0.01
**Chemical composition** ^3^			
Crude protein, %	17.20		
Ether extract, %	4.30		
Crude fiber, %	3.50		
Ash, %	5.00		
Sodium, %	0.20		
Calcium, %	0.50		
Phosphorus, %	0.60		
Lysine, %	1.30		
Methionine, %	0.50		
Net Energy (NE), kcal/kg	2340		

^1^ Premix contained the following per kg nutrients of the diet: 6500.00 IU vitamin A, 1200.00 IU vitamin D3 (cholecalciferol), 47.50 mg betaine anhydrous, 40.0 mg betaine hydrochloride, 0.2 mg biotin, 0.50 mg folic acid, 20.0 mg niacinamide, 10.0 mg calcium pantothenate, 10.0 mg vitamin B1, 0.03 mg vitamin B12 (cobalamin), 5.0 mg vitamin B2 (riboflavin), 2.0 mg vitamin B6 (pyridoxine hydrochloride), 40 mg all-rac-atocopheryl-acetate; 1.5 mg vitamin K3, 15.0 mg Cu, 0.50 mg I, 40.0 mg Mn, 0.25 mg Se, 70.0 mg Zn oxide, 4680.00 L-lysine, 1970.00 mg L-threonine, 384.0 mg L-tryptophan. ^2^ Vitamin E provided per kilogram of premix: 485 mg. ^3^ Nutrient (expressed as fed basis) and net energy content were calculated using Plurimix software (Fabermatica, CR, Italy).

**Table 2:** Growth performance of piglets fed with different dietary polyphenol sources.

Groups	CON^−^	CON^+^	PP1	PP2	PP3	PP4	SEM	*p*-Value
BW (d0), kg	7.03	7.14	7.24	7.12	7.23	7.34	0.07	ns
BW (d35), kg	18.94 ^a^	19.55 ^a^	15.04 ^b^	19.08 ^a^	19.98 ^a^	19.72 ^a^	0.23	<0.05
ADG, g/d	340 ^a^	354 ^a^	223 ^b^	342 ^a^	364 ^a^	354 ^a^	0.005	<0.05
FCR, kg/kg	1.78 ^a^	1.77 ^a^	2.12 ^b^	1.79 ^a^	1.76 ^a^	1.73 ^a^	-	<0.05
Mortality, %	0	0	0	0	0	0	-	-

BW: body weight; ADG; average daily gain; FCR; feed conversion ratio; SEM: standard error of the mean ^a,b^. Means within a row with different letters are significantly different at *p* < 0.05.

**Table 3:** Effect of dietary treatment (D) and sampling time (T) on blood metabolites.

Groups	CON^−^	CON^+^	PP1	PP2	PP3	PP4	SEM	Diet	Treatment
Vit. A (ug/L)									
d 0	304.8	387.3	345.4	347.0	304.2	382.7	10.2		
d 35	310.3	353.6	395.4	329.6	308.0	337.3	9.35	0.045	ns
Vit. E * (mg/L)									
d 0	0.71	0.79	0.93	0.97	0.88	0.67	0.053		
d35	1.12	2.12	1.00	0.96	1.46	1.51	0.080	0.072	<0.001
IgG (mg/mL)									
d 0	1.57	2.15	2.45	1.98	2.42	1.88	0.09		
d 35	2.97	2.63	2.83	2.38	3.28	2.26	0.121	ns	0.001
IL-6 (ng/mL)									
d 0	0.13	0.01	0.45	0.06	0.05	0.05	0.017		
d35	0.04	0.04	0.04	0.04	0.11	0.05	0.013	ns	ns
IL-8 (ng/mL)									
d 0	0.302	0.201	0.181	0.282	0.354	0.312	0.022		
d35	0.708	0.724	0.787	0.390	0.404	0.358	0.038	0.022	0.001
IL-10 (ng/mL)									
d 0	0.495	0.108	0.158	0.281	0.204	0.279	0.06		
d 35	0.288	0.376	0.208	0.234	0.304	0.540	0.096	ns	ns
IFN-γ (ng mL)									
d 0	1.31	1.03	0.94	1.19	0.87	0.85	0.066		
d 35	0.55	0.54	1.34	0.54	1.20	0.65	0.120	ns	<0.001
TNF-α * (ng mL)									
d 0	0.019	0.015	0.012	0.005	0.010	0.031	0.000		
d35 **	0.003	0.000	0.001	0.003	0.004	0.009	0.000	ns	<0.01

SEM: standard error of the mean; * *p*-value calculated as ln-transformed; ** Although some pigs had values above the technique cut-off, the mean value was below the cut-off.

**Table 4:** Effect of dietary treatment (D) and sampling time (T) on antioxidant defenses.

	CON^-^	CON^+^	PP1	PP2	PP3	PP4	D	T	DxT ^#^
GPx	(U/mL)								
d 0	1.13 ± 0.19	0.99 ± 0.17	0.96 ± 0.20	1.13 ± 0.30	1.31 ± 0.30	1.24 ± 0.23	0.001	<0.001	<0.001
d 35	1.51 ± 0.26	1.34 ± 0.22	2.33 ± 0.39	1.42 ± 0.20	1.38 ± 0.32	1.44 ± 0.44			
SOD	(U/mL)								
d 0	24.2 ± 8.2	24.2 ± 10.5	20.8 ± 9.7	14.4 ± 2.0	28.1 ± 14.6	17.3 ± 7.7	0.112	<0.001	0.003
d35	10.5 ± 4.0	12.1 ± 6.8	16.1 ± 8.7	16.1 ± 6.0	19.1 ± 7.9	19.8 ± 6.5			
KRL_WB_ *	(min)								
d 0	125.4 ± 15.7	126.7 ± 8.5	125.9 ± 13.2	122.1 ± 16.9	134.4 ± 17.6	117.4 ± 15.0	0.033	<0.001	ns
d 35	105.8 ± 13.3	125.3 ± 17.7	106.2 ± 10.6	112.6 ± 17.2	120.9 ± 16.6	111.5 ± 9.9			
KRL_RBC_ **	(min)								
d 0	107.6 ± 17.1	107.5 ± 13.1	107.3 ± 17.4	96.0 ± 12.1	104.3 ± 17.7	93.6 ± 16.9	ns	ns	ns
d35	94.6 ± 18.1	102.1 ± 19.1	102.9 ± 10.6	101.3 ± 17.5	104.3 ± 17.3	97.3 ± 11.7			

* KRL_WB_: KRL half-hemolysis time on whole blood; ** KRL_RBC_: KRL half-hemolysis time on red blood cells. Results are expressed by means ± standard deviations of N = 10 piglets in each group. *^#^ p*-values of repeated measures ANOVA: interactions between dietary treatment (D) and sampling time (T) main effects.

## Data Availability

The data presented in the current study are available from the corresponding authors on reasonable request.

## References

[1] Liao S., Omage S.O., Börmel L., Kluge S., Schubert M., Wallert M., Lorkowski S. (2022). Vitamin E and metabolic health: Relevance of interactions with other micronutrients. Antioxidants.

[2] Lee G.Y., Han S.N. (2018). The role of vitamin E in immunity. Nutrients.

[3] Håkansson J., Hakkarainen J., Lundeheim N. (2001). Variation in vitamin E, glutathione perroxidase and retinol concentrations in blood plasma of primiparous sows and their piglets, and in vitamin E, selenium and retinol contents in sows’ milk. Acta Agric. Scand. Sect. A Anim. Sci..

[4] Meyer W.R., Mahan D.C., Moxon A.L. (1981). Value of dietary selenium and vitamin E for weanling swine as measured by performance and tissue selenium and glutathione peroxidase activities. J. Anim. Sci..

[5] Mahan D.C., Lepine A.J. (1991). Effect of pig weaning weight and associated nursery feeding programs on subsequent performance to 105 kilograms body weight. J. Anim. Sci..

[6] Lauridsen C., Jensen S.K. (2005). Influence of supplementation of all-rac-α-tocopheryl acetate preweaning and vitamin C postweaning on α-tocopherol and immune responses of piglets. J. Anim. Sci..

[7] Rey A.I., López-Bote C.J., Litta G. (2017). Effects of dietary vitamin E (DL-α-tocopheryl acetate) and vitamin C combination on piglets oxidative status and immune response at weaning. J. Anim. Feed Sci..

[8] National Research Council (2012). Nutrient Requirements of Swine.

[9] Orengo J., Hernández F., Martínez-Miró S., Sánchez C.J., Peres Rubio C., Madrid J. (2021). Effects of commercial antioxidants in feed on growth performance and oxidative stress status of weaned piglets. Animals.

[10] Serra V., Salvatori G., Pastorelli G. (2021). Dietary polyphenol supplementation in food producing animals: Effects on the quality of derived products. Animals.

[11] Brenes A., Viveros A., Chamorro S., Arija I. (2016). Use of polyphenol-rich grape by-products in monogastric nutrition. A review. Anim. Feed. Sci. Tech..

[12] Lipiński K., Mazur M., Antoszkiewicz Z., Purwin C. (2017). Polyphenols in monogastric nutrition—A review. Ann. Anim. Sci..

[13] Surai P.F. (2014). Polyphenol compounds in the chicken/animal diet: From the past to the future. J. Anim. Physiol. Anim. Nutr..

[14] Landete J.M. (2013). Dietary intake of natural antioxidants: Vitamins and polyphenols. Crit. Rev. Food. Sci. Nutr..

[15] Gan Z., Wei W., Li Y., Wu J., Zhao Y., Zhang L., Wang T., Zhong X. (2019). Curcumin and resveratrol regulate intestinal bacteria and alleviate intestinal inflammation in weaned piglets. Molecules.

[16] Wang R., Yu H., Fang H., Jin Y., Zhao Y., Shen J., Zhou C., Li R., Wang J., Fu Y. (2020). Effects of dietary grape pomace on the intestinal microbiota and growth performance of weaned piglets. Arch. Anim. Nutr..

[17] Lu Y., Zhao M., Mo J., Lan G., Liang J. (2022). Dietary supplementation ellagic acid on the growth, intestinal immune response, microbiota, and inflammation in weaned piglets. Front. Vet. Sci..

[18] Galassi G., Battelli M., Verdile N., Rapetti L., Zanchi R., Arcuri S., Petrera F., Abeni F., Crovetto G.M. (2021). Effect of a polyphenol-based additive in pig diets in the early stages of growth. Animals.

[19] Legislative Decree 26/2014—Implementation of Directive 2010/63/EU on the Protection of Animals Used for Scientific Purposes; GU n. 61 of 14 March 2014. https://www.gazzettaufficiale.it/eli/id/2014/03/14/14G00036/sg.

[20] Directive 2010/63/EU of the European Parliament and of the Council of 22 September 2010 on the Protection of Animals Used for Scientific Purposes. OJ L276/33-79. https://eur-lex.europa.eu/legal-content/EN/TXT/PDF/?uri=CELEX:32010L0063&from=EN.

[21] Schüep W., Rettenmaier R. (1994). Analysis of vitamin E homologs in plasma and tissue: High-performance liquid chromatography. Methods Enzymol..

[22] Simoni M., Goi A., Pellattiero E., Mavrommatis A., Tsiplakou E., Righi F., De Marchi M., Manuelian C.L. (2022). Long-term administration of a commercial supplement enriched with bioactive compounds does not affect feed intake, health status, and growth performances in beef cattle. Arch. Anim. Breed..

[23] Khalifian S., Raimondi G., Brandacher G. (2015). The use of luminex assays to measure cytokines. J. Invest. Dermatol..

[24] Prost M. (2003). Method for Determining the Antiradical Defense Potential and Use Thereof, in Particular in Veterinary and Human Preventive Therapeutics. U.S. Patent.

[25] Pastorelli G., Faustini M., Corino C., Rossi R. (2013). Kit Radicaux Libres, a biological application for monitoring oxidative stress in pigs. Ital. J. Anim. Sci..

[26] De Bellis R., Piacentini M.P., Meli M.A., Mattioli M., Menotta M., Mari M., Valentini L., Palomba L., Desideri D., Chiarantini L. (2019). In vitro effects on calcium oxalate crystallization kinetics and crystal morphology of an aqueous extract from Ceterach officinarum. Analysis of a potential antilithiatic mechanism. PLoS ONE.

[27] Garrett A.R., Murray B.K., Robison R.A., O’Neill K.L. (2010). Measuring antioxidant capacity using the ORAC and TOSC assays. Methods Mol. Biol..

[28] Fundo J.F., Miller F.A., Garcia E., Santos J.R., Silva C.L., Brandão T.R. (2018). Physicochemical characteristics, bioactive compounds and antioxidant activity in juice, pulp, peel and seeds of Cantaloupe melon. J. Food Meas. Charact..

[29] Costa L.G., Tait L., de Laat R., Dao K., Giordano G., Pellacani C., Cole T.B., Furlong C.E. (2013). Modulation of paraoxonase 2 (PON2) in mouse brain by the polyphenol quercetin: A mechanism of neuroprotection?. Neurochem. Res..

[30] Dhama K., Tiwari R., Chakraborty S., Saminathan M., Kumar A., Karthik K., Wani M.Y., Singh S.V., Rahal A. (2014). Evidence based antibacterial potentials of medicinal plants and herbs countering bacterial pathogens especially in the era of emerging drug resistance: An integrated update. Int. J. Pharmacol..

[31] Christaki E., Giannenas I., Bonos E., Florou-Paneri P., Florou-Paneri P., Christaki E., Giannenas I. (2020). Chapter 2-Innovative uses of aromatic plants as natural supplements in nutrition. Feed Additives.

[32] Hasegawa S., Miyake M. (1996). Biochemistry and biological functions of citrus limonoids. Food Rev. Int..

[33] Drewnowski A., Henderson S.A., Shore A.B. (1997). Taste responses to naringin, a flavonoid, and the acceptance of grapefruit juice are related to genetic sensitivity to 6-n-propylthiouracil. Am. J. Clin. Nutr..

[34] Zhang H.J., Jiang X.R., Mantovani G., Lumbreras A.E.V., Comi M., Alborali G., Savoini G., Dell’Orto V., Bontempo V. (2014). Modulation of plasma antioxidant activity in weaned piglets by plant polyphenols. Ital. J. Anim. Sci..

[35] Gessner D.K., Fiesel A., Most E., Dinges J., Wen G., Ringseis R., Eder K. (2013). Supplementation of a grape seed and grape marc meal extract decreases activities of the oxidative stress-responsive transcription factors NF-κB and Nrf2 in the duodenal mucosa of pigs. Acta Vet. Scand..

[36] Rajković E., Schwarz C., Tischler D., Schedle K., Reisinger N., Emsenhuber C., Ocelova V., Roth N., Frieten D., Dusel G. (2021). Potential of grape extract in comparison with therapeutic dosage of antibiotics in weaning piglets: Effects on performance, digestibility and microbial metabolites of the ileum and colon. Animals.

[37] Navarrete M., Rangel C., Espinosa-García J., Corchado J.C. (2005). Theoretical study of the antioxidant activity of vitamin E: Reactions of α-tocopherol with the hydroperoxy radical. J. Chem. Theory Comput..

[38] Dao D.Q., Ngo T.C., Thong N.M., Nam P.C. (2017). Is Vitamin A an Antioxidant or a Pro-oxidant?. J. Phys. Chem. B.

[39] Corbi G., Conti V., Komici K., Manzo V., Filippelli A., Palazzo M., Vizzari F., Davinelli S., Di Costanzo A., Scapagnini G. (2018). Phenolic plant extracts induce sirt1 activity and increase antioxidant levels in the rabbit’s heart and liver. Oxid. Med. Cell. Longev..

[40] Corino C., Oriani G., Pantaleo L., Pastorelli G., Salvatori G. (1999). Influence of dietary vitamin E supplementation on “heavy” pig carcass characteristics, meat quality, and vitamin E status. J. Anim. Sci..

[41] Lauridsen C. (2010). Evaluation of the effect of increasing dietary vitamin E in combination with different fat sources on performance, humoral immune responses and antioxidant status of weaned pigs. Anim. Feed Sci. Technol..

[42] Song R., Chen C., Johnston L.J., Kerr B.J., Weber T.E., Shurson G.C. (2014). Effects of feeding diets containing highly peroxidized distillers dried grains with solubles and increasing vitamin E levels to wean–finish pigs on growth performance, carcass characteristics, and pork fat composition. J. Anim. Sci..

[43] Wang D., Jang Y.D., Kelley M., Rentfrow G.K., Azain M.J., Lindemann M.D. (2023). Effects of multiple vitamin E levels and two fat sources in diets for swine fed to heavy slaughter weight of 150 kg: II. Tissue fatty acid profile, vitamin E concentrations, immune capacity, and antioxidant capacity of plasma and tissue. Transl. Anim. Sci..

[44] Han X., Shen T., Lou H. (2007). Dietary polyphenols and their biological significance. Int. J. Mol. Sci..

[45] Faria A., Fernandes I., Norberto S., Mateus N., Calhau C. (2014). Interplay between anthocyanins and gut microbiota. J. Agric. Food Chem..

[46] Gessner D.K., Ringseis R., Eder K. (2017). Potential of plant polyphenols to combat oxidative stress and inflammatory processes in farm animals. J. Anim. Physiol. Anim. Nutr..

[47] Hackman R.M., Polagruto J.A., Zhu Q.Y., Sun B., Fujii H., Keen C.L. (2008). Flavanols: Digestion, absorption and bioactivity. Phytochem. Rev..

[48] Hein E.M., Rose K., van’t Slot G., Friedrich A.W., Humpf H.U. (2008). Deconjugation and degradation of flavonol glycosides by pig cecal microbiota characterized by fluorescence in situ hybridization (FISH). J. Agric. Food Chem..

[49] Moreira I., Mahan D.C. (2002). Effect of dietary levels of vitamin E (all-rac-tocopheryl acetate) with or without added fat on weanling pig performance and tissue α-tocopherol concentration. J. Anim. Sci..

[50] Sivertsen T., Vie E., Bernhoft A., Baustad B. (2007). Vitamin E and selenium plasma concentrations in weanling pigs under field conditions in Norwegian pig herds. Acta Vet. Scand..

[51] Hinson R.B., McCormick K.A., Moser R.L., Ackerman M.A., Main R.G., Mahoney J.A. (2022). Reduced vitamin supplementation with fat-soluble vitamins A, D, and E added at National Research Council requirements may not be adequate for optimal sow and progeny performance. J. Swine Health Prod..

[52] Chung Y.K., Mahan D.C., Lepine A.J. (1992). Efficacy of dietary D-α-tocopherol and DL-α-tocopheryl acetate for weanling pigs. J. Anim. Sci..

[53] Liu P., Kerr B.J., Weber T.E., Chen C., Johnston L.J., Shurson G.C. (2014). Influence of thermally oxidized vegetable oils and animal fats on intestinal barrier function and immune variables in young pigs. J. Anim. Sci..

[54] García G.R., Dogi C.A., Ashworth G.E., Berardo D., Godoy G., Cavaglieri L.R., De Moreno de LeBlanc A., Greco C.R. (2016). Effect of breast feeding time on physiological, immunological and microbial parameters of weaned piglets in an intensive breeding farm. Vet. Immunol. Immunopathol..

[55] Moeser A.J., Pohl C.S., Rajput M. (2017). Weaning stress and gastrointestinal barrier development: Implications for lifelong gut health in pigs. Anim. Nutr..

[56] Rooke J.A., Carranca C., Bland I.M., Sinclair A.G., Ewen M., Bland V.C., Edwards S.A. (2003). Relationships between passive absorption of immunoglobulin G by the piglet and plasma concentrations of immunoglobulin G at weaning. Livest. Prod. Sci..

[57] de Groot N., Fariñas F., Cabrera-Gómez C.G., Pallares F.J., Ramis G. (2021). Weaning causes a prolonged but transient change in immune gene expression in the intestine of piglets. J. Anim. Sci..

[58] Pistol G.C., Palade L.M., Marin D.E., Stancu M., Taranu I. (2019). The effect of grape wastes, wine industry byproducts, on inflammatory and antioxidant biomarkers in post-weaning piglets. Sci. Pap. Anim. Sci. Ser. Lucr. Ştiinţifice-Ser. Zooteh..

[59] Cao S.T., Wang C.C., Wu H., Zhang Q.H., Jiao L.F., Hu C.H. (2018). Weaning disrupts intestinal antioxidant status, impairs intestinal barrier and mitochondrial function, and triggers mitophagy in piglets. J. Anim. Sci..

[60] Li Y., Zhao X., Jiang X., Chen L., Hong L., Zhuo Y., Lin Y., Fang Z., Che L., Feng B. (2020). Effects of dietary supplementation with exogenous catalase on growth performance, oxidative stress, and hepatic apoptosis in weaned piglets challenged with lipopolysaccharide. J. Anim. Sci..

[61] Zheng P., Yu B., He J., Tian G., Luo Y., Mao X., Zhang K., Che L., Chen D. (2013). Protective effects of dietary arginine supplementation against oxidative stress in weaned piglets. Br. J. Nutr..

[62] Xiong S., Guo R., Yang Z., Xu L., Du L., Li R., Xiao F., Wang Q., Zhu M., Pan X. (2015). Treg depletion attenuates irradiation-induced pulmonary fibrosis by reducing fibrocyte accumulation, inducing Th17 response, and shifting IFN-γ, IL-12/IL-4, IL-5 balance. Immunobiology.

[63] Lauridsen C. (2019). From oxidative stress to inflammation: Redox balance and immune system. Poult. Sci..

[64] Al-Sadi R., Boivin M., Ma T. (2009). Mechanism of cytokine modulation of epithelial tight junction barrier. Front. Biosci..

[65] Opal S.M., DePalo V.A. (2000). Anti-inflammatory cytokines. Chest.

[66] Silva-Guillen Y.V., Arellano C., Boyd R.D., Martinez G., Van Heugten E. (2020). Growth performance, oxidative stress and immune status of newly weaned pigs fed peroxidized lipids with or without supplemental vitamin E or polyphenols. J. Anim. Sci Biotechnol..

[67] Fiesel A., Gessner D.K., Most E., Eder K. (2014). Effects of dietary polyphenol-rich plant products from grape or hop on pro-inflammatory gene expression in the intestine, nutrient digestibility and faecal microbiota of weaned pigs. BMC Vet. Res..

[68] Guo M., Wang H., Xu S., Zhuang Y., An J., Su C., Liu X. (2020). Alteration in gut microbiota is associated with dysregulation of cytokines and glucocorticoid therapy in systemic lupus erythematosus. Gut Microbes.

[69] Laskowska E., Jarosz Ł., Grądzki Z. (2017). The effect of feed supplementation with effective microorganisms (EM) on pro-and anti-inflammatory cytokine concentrations in pigs. Res. Vet. Sci..

[70] Pomorska-Mól M., Wierzchosławski K., Włodarek J., Gogulski M., Pejsak Z. (2020). Dynamics of pro-and anti-inflammatory cytokine changes in serum and assessment of their diagnostic utility during lactation impairment in pigs. Res. Vet. Sci..

[71] Turin L., Torinesi R., Pastorelli G. (2019). Real-time PCR detection of the effect of postweaning on the expression of cytokines and NF-kB in piglets. J. Biol. Regul. Homeost. Agents.

[72] Rossi R., Pastorelli G., Corino C. (2013). Application of KRL test to assess total antioxidant activity in pigs: Sensitivity to dietary antioxidants. Res. Vet. Sci..

[73] Mao X., Lv M., Yu B., He J., Zheng P., Yu J., Wang Q., Chen D. (2014). The effect of dietary tryptophan levels on oxidative stress of liver induced by diquat in weaned piglets. J. Anim. Sci. Biotechnol..

[74] Han H., Liu Z., Yin J., Gao J., He L., Wang C., Hou R., He X., Wang G., Li T. (2021). D-galactose induces chronic oxidative stress and alters gut microbiota in weaned piglets. Front. Physiol..

[75] Degroote J., Vergauwen H., Wang W., Van Ginneken C., De Smet S., Michiels J. (2020). Changes of the glutathione redox system during the weaning transition in piglets, in relation to small intestinal morphology and barrier function. J. Anim. Sci. Biotechnol..

